# Fair publication of qualitative research in health systems: a call by health policy and systems researchers

**DOI:** 10.1186/s12939-016-0368-y

**Published:** 2016-06-22

**Authors:** Social science approaches for research and engagement in health policy & systems (SHaPeS) thematic working group of Health Systems Global, Regional Network for Equity in Health in East and Southern Africa (EQUINET), and Emerging Voices for Global Health, Karen Daniels, Rene Loewenson, Asha George, Natasha Howard, Gergana Koleva, Simon Lewin, Bruno Marchal, Devaki Nambiar, Ligia Paina, Emma Sacks, Kabir Sheikh, Moses Tetui, Sally Theobald, Stephanie M. Topp, Anthony B. Zwi

**Affiliations:** Health Systems Research Unit, South African Medical Research Council, Cape Town, Western Cape South Africa; Health Policy and Systems Division, School of Public Health and Family Medicine, University of Cape Town, Cape Town, South Africa; Training and Research Support Centre, Regional Network for Equity in Health in East and Southern Africa (EQUINET), Harare, East and Southern Africa; School of Public Health, University of the Western Cape, Western Cape, South Africa; Department of International Health, Johns Hopkins University School of Public Health, Baltimore, USA; London School of Hygiene & Tropical Medicine, London, UK; Patient Experience Researcher and Advocate for Patient and Public Involvement, Sofia, Bulgaria; Global Health Unit, Knowledge Centre for the Health Services, Norwegian Institute of Public Health, Oslo, Norway; Department of Public Health, Institute of Tropical Medicine, Antwerp, Belgium; Public Health Researcher, New Delhi, India; Department of International Health, Johns Hopkins School of Public Health, Baltimore, USA; USAID Maternal and Child Survival Program (MCSP)/ICF International, Baltimore, USA; Public Health Foundation of India, New Delhi, India; Makerere University School of Public Health, Makerere, Uganda; Umea International School Of Public Health, Umea University, Umea, Sweden; Department of International Public Health, Liverpool School of Tropical Medicine, Liverpool, UK; Institute of Development Studies, Sussex, UK; College of Public Health, Medical and Veterinary Science, James Cook University, Townsville City, Australia; Health Rights and Development, School of Social Sciences, The University of New South Wales, New South Wales, Australia

An open letter from Trisha Greenhalgh et al. [1] to the editors of the British Medical Journal (BMJ) triggered wide debate by health policy and systems researchers (HPSRs) globally on the inadequate recognition of the value of qualitative research and the resulting deficit in publishing papers reporting on qualitative research [2]. One key dimension of equity in health is that researchers are able to disseminate their findings and that they are taken into account in a fair and just manner, so that they can inform health policy and programmes. The Greenhalgh et al. letter and editorial responses [3, 4] were actively discussed within “SHAPES”, a thematic group within Health Systems Global, focused on Social Science approaches for research and engagement in health policy & systems (http://healthsystemsglobal.org/twg-group/6/Social-science-approaches-for-research-and-engagement-in-health-policy-amp-systems/) and within EQUINET, a regional network working on health equity research in East and Southern Africa (www.equinetafrica.org). Our discussion precipitated in this follow up open letter/commentary, which has 170 co-signatories. Collectively, we feel that barriers to publication of qualitative research limit publication of many exemplary studies, and their contribution to understanding important dimensions of health care, services, policies and systems.

While we work on different aspects of health systems, we *all* feel that more serious recognition of the value of qualitative research is required, including to disseminate evidence and contribute voice to advance equity in health. In the spirit of collective engagement for research excellence that makes a difference to the communities and systems with which we work, we add our voices to this debate. We are particularly disenchanted by our general experience of the limited and often inadequate publication of qualitative research in the major health and medical journals, and the resultant loss of important insights for those working in, or concerned with, health services and systems, including around clinical decision-making.

The editors of one major medical journal have, for example, asserted a desire to publish “*studies with more definitive—not exploratory—research questions that are relevant to an international audience and that are most likely to change clinical practice and help doctors make better decisions*” [4]. Even for medical journals, this reinforces a somewhat narrow view of health care, and of the forms of evidence relevant to clinical decision making [5]. Restricting publication to quantitative research risks marginalising important bodies of knowledge such as those concerned with the social nature of health and illness or the way in which service providers incorporate (or neglect) such knowledge. Given the complex nature of health, policy, services and systems, we would argue that more inclusive and wide ranging insights and perspectives -- rather than fragmented ones -- are required, and methodologies should not be limited to quantitative approaches [6, 7]. Doctors do not (and should not) make decisions about patients in a vacuum, but operate within the broader social, political and systemic contexts of health care. Appreciating the nature of such influences can assist in making better informed and more appropriate decisions, especially given that such influences are rarely only ‘technical’.

Given the varied influences on clinical practice, Health Systems Global has observed that such decisions must take account of relationships and complexity in health systems, and cannot be addressed through simple causality models [8]. Qualitative social science approaches have a key role in uncovering these broader relationships and complexities, and can crucially inform decision making by providing them with necessary insights and engaging them in these dynamics. A greater appreciation of the value of qualitative approaches in the study of health care systems and policy can only improve decision-making in our age of high political consciousness and rapid information availability.

Working with the World Health Organization (WHO) in developing guidance and policies for maternal health, for instance, HSG members have found a demand from policy makers, planners, and implementers for qualitative research to inform decisions on health systems and clinical recommendations and to identify implementation considerations. Important evidence has been derived from both systematic reviews of qualitative studies and mixed-methods reviews, for example on the mistreatment of women in health facilities during childbirth [9] and concerning barriers and facilitators to task-shifting in midwifery services [10] and for lay health workers [11]. These reviews have been used in guideline development and have also stimulated wider public interest and debate, as in the case of the Bohren review, which was documented in the New York Times [12]. The WHO Handbook for Guideline Development recognises the importance of such evidence and includes a chapter on using qualitative research in developing guidelines [13]. The WHO and a number of other agencies have also supported the development of a new approach to assess how much confidence to place in evidence from systematic reviews of qualitative studies [14], to facilitate using such evidence to inform health care decisions. This experience highlights the crucial roles that qualitative evidence syntheses can play in gathering qualitative evidence addressing a health question, developing new insights and theory from this evidence to inform policy and practice (including clinical practice), and identifying research gaps. The major health and medical journals should encourage the submission of such syntheses in the same way that many encourage submission of systematic reviews of the effectiveness of health interventions and of diagnostic tests.

Decisions concerning health systems and medical practice globally are taken by a range of professionals, not only or even primarily, by doctors. Multidisciplinary teams play a key role in promoting more holistic equitable models of care, and other providers are crucial in those many parts of the world where there are no or not enough doctors. Here, many decisions need to be taken by wider health care teams and clients equally involved in health promotion and care plans. ‘Biopsychosocial’ models [of care] correctly identify disorders as outcomes of interactions between biological, psychological and social determinants. Qualitative research demonstrates the subjectivity of health workers in the therapeutic alliance, relationships and communication between health workers and clients [15–17], and the role of social literacy and of people’s values, preferences, and lifestyles in medical decision-making process when assessing the merits of various treatment alternatives for specific health problems [18]. The expanding literature on ‘person centred care’ recognises these issues.

Qualitative research facilitates examination of quality of interaction [19] and identifies the patient as an individual (and member of a family and community) experiencing care rather than being the subject of a disease process [17]. Mixed methods help identify and explain the factors that influence outcomes, and important dimensions of care, such as trust and social support, not all of which can be ‘measured’ by numbers alone [17]. Qualitative research also facilitates better understanding of the political and social determinants of care [20] including gender, social literacy, values, preferences [18, 21, 22]. One area where understanding these complex dynamics is pertinent, is with the deploying of lay/community health workers [23], especially since they work so close to communities [18, 24]. Despite important randomised controlled trials on the effectiveness of lay/community health workers, and a systematic review of these trials [25], the review authors argue that qualitative research is still needed to explain the complexities of the review findings [26]. This is echoed by one of our signatories:

*“I do love working on numbers …, but I can only understand my findings and know how to model my data if I do have a clearer picture of the context and only after understanding the qualitative work. The latter facilitates my understanding beyond what the numbers show”.* (Erlyn Rachelle Macarayan, *an Emerging Voice for Global Health [*http://www.ev4gh.net/] *Philippines).*

Participatory action research (PAR) is one form of qualitative research that has provided voice in research for marginalised groups and produced new evidence on risk-health patterning that has contributed to declines in work related ill-health and injuries [27–29]. This approach has also contributed evidence on environmental determinants of health; barriers and enablers in managing ill health; and on learning about the roles and social relationships contributing to effective prevention and care [30].

In health service decision making, anthropological and qualitative studies have elucidated citizen responses to insurance, including why people enrol or drop out, and how families use health insurance [31, 32]. In relation to the Ebola crisis, this kind of research is particularly important to overcome implementation and coverage deficits, and to address the gap between policy intention and policy in practice [33, 34], to illuminate issues around trust and health service utilisation, and may contribute to building future health systems resilience [35].

The methodological diversity in qualitative research not only generates new evidence and knowledge for health systems policy, planning and practice, but also incorporates approaches to engage and participate with communities (and users of services) to utilise evidence and solutions to create change [30]. PAR and implementation research [30] embrace change through the co-creation of research with a range of stakeholders. For example, Othieno and colleagues [36] worked with women from low income communities in designing and implementing community mental health services [36]. Other examples of change and learning from change embedded within research, have been documented [30]. The methods systematize local experience and synthesize collective analysis on relationships and causes of problems.

The reflexive process is directly linked to new knowledge and action, influenced by understanding of history, culture, and local context and embedded in social relationships. In narrowly defining what research and thus what knowledge counts as important, the opportunity to learn from this richness is lost. Furthermore, this effectively silences the voices of community members, particularly those who are marginalised across all countries [37–40].

Such research belongs in mainstream publication on health and should not simply be assigned to ‘special interest journals’. Doing so risks devaluing work relevant to health services, weakens understanding of the interface between qualitative and quantitative research and undermines the breadth and quality of analysis. Furthermore, the perception that such work will not be considered, even before peer review, has already resulted in some of our members sending manuscripts to special interest journals, and consequently having it hidden from a more general audience who might have benefitted from its insights.

The inadequate publication space for qualitative studies is a generalised problem that undermines our understanding of and response to health system challenges. Addressing it calls for specific strategies, such as establishing panels of suitable reviewers and enhancing the quality of guidelines for researchers and authors.

We have cited numerous examples of the contribution of qualitative research to health service decision-making. It is problematic to reinforce the dichotomy between qualitative and quantitative research. Qualitative studies provide evidence that informs health services decision making, deepens interpretation and understanding, and assists all to better deal with the complexity inherent in health problems and the search for their solutions. Qualitative insights will also assist in closing the policy-implementation gap. We look forward to further debate and publication, including in this journal, on approaches to overcoming barriers to publication of qualitative research on health policy and systems, for researchers and journals, to ensure that crucial domains of research and knowledge are not excluded from health systems policy and practice.

## Signatories

Social science approaches for research and engagement in health policy & systems (SHaPeS) thematic working group of Health Systems Global, 

Regional Network for Equity in Health in East and Southern Africa (EQUINET),

and Emerging Voices for Global Health.

## Co-signatories (in alphabetical order).

1. Abdelwahab, Jalaa; Health Specialist, UNICEF, United States of America
2. Abramowitz, Sharon; PhD. Medical Anthropologist
3. Akhurst, Jacqueline; Associate Professor; Psychology Department, Rhodes University, South Africa.
4. Arhinful, Daniel K.; Research Fellow, Noguchi Memorial Institute for Medical Research, University of Ghana, Ghana
5. Atkins, Salla; Project manager, School of Health Sciences, University of Tampere, Affiliated researcher, Department of Public Health, Karolinska Institutet, Finland and Sweden
6. Ba, Maymouna; Researcher, Senegal
7. Baba Dieu Merci, Amuda; Executive Director, IPASC, Democratic Republic of Congo
8. Balabanova, Dina; Senior in Lecturer Health Systems/Policy, London School Hygiene Tropical Medicine, United Kingdom
9. Bennett, Sara; Associate Professor, Johns Hopkins Bloomberg School of Public Health, United States of America
10.  Bhana, Arvin; Chief Specialist Scientist, Health Systems Research Unit, South Africa Medical Research Council, South Africa
11.  Birungi, Charles;, Institute for Global Health, University College London, Country: United Kingdom
12.  Black, Gill; Director and Head of Health Participation, Sustainable Livelihoods Foundation, South Africa
13.  Bose, Shibaji; Consultant, Research Influence Policy Uptake - Future Health Systems, STEPS Centre, Norway, University of Life Sciences, India
14.  Boulle, Therese; Researcher, University of Cape Town, South Africa
15.  Bozorgmehr, Kayvan; Senior Research Fellow, Dept. of General Practice & Health Services Research, University Hospital Heidelberg, Germany
16.  Bradley, Hazel; Senior Lecturer/Snr Academic Programme Co-ordinator School of Public Health, University of the Western Cape, South Africa
17.  Bruen, Carlos; Assistant Director, Academic Affairs, SPHeRE Programme, Division of Population Health Sciences, Royal College of Surgeons in Ireland, Ireland
18.  Brugha, Ruairi; Head of Department. Department of Epidemiology & Public Health Medicine, Royal College of Surgeons in Ireland, Ireland
19.  Burman, Chris, DevFTI/Rural Development and Innovation Hub, University of Limpopo, South Africa
20.  Byrne, Elaine; Senior Lecturer, Institute of Leadership, Royal College of Surgeons in Ireland, Ireland
21.  Byskov, Jens; Emeritus, Faculty of Health and Medical Sciences, University of Copenhagen, Denmark - and Research and Health Systems Technical Advisor, Department of Public Health, Medical School, University of Zambia, Zambia
22.  Carlsen, Benedicte; Research professor, Uni Research Rokkan Centre, Norway
23.  Chakravarty, Aruna Bhattacharya, PhD (Anthropology), Associate Professor, Indian institute of Public Health – Delhi, Public Health Foundation of India, India
24.  Chikaphupha, Kingsley Rex; Researcher, Institution: Research for Equity and Community Health (REACH) Trust, Malawi
25.  Choonara, Shakira; PhD Fellow, Centre for Health Policy, University of the Witwatersrand, South Africa
26.  Coe, Anna-Britt; Associate Professor, Epidemiology and Global Health Unit, Dept. Of Public Health and Clinical Medicine, Umeå University, Sweden
27.  Colombini, Manuela; Lecturer, London School of Hygiene and Tropical Medicine, South Africa
28.  Colvin, Christopher J.; Assoc. Profesor & Division Head, Division of Social and Behavioural Sciences, Co-Director, South African Social Science and HIV (SASH) Programme, Member, Centre for Infectious Disease Epidemiology and Research (CIDER), School of Public Health and Family Medicine, University of Cape Town
29.  Criel, Bart: Professor, Head of the Equity & Health Unit, Department of Public Health, Institute of Tropical Medicine, Belgium
30.  Cubaka Vincent; Kalumire, Center for Global Health, Department of Public Health, Aarhus University, Denmark, Department of Primary Health Care, School of medicine and Pharmacy, University of Rwanda, Rwanda
31.  D’Ambruoso, Lucia; Lecturer in Global Health, Institute of Applied Health Sciences, University of Aberdeen, Scotland United Kingdom
32.  Daniels, Karen; Specialist Scientist, Health Systems Research Unit, South African Medical Research Council, Honorary Senior Lecture, Health Policy and Systems Division, School of Public Health and Family Medicine, University of Cape Town, South Africa
33.  Daviaud, Emmanuelle; Senior Specialist Scientist, Health Systems Research Unit, South African Medical Research Council, South Africa
34.  de koning, Korrie; Social Scientist, Independent Consultant and Associate of The Royal Tropical Institute (KIT), The Netherlands
35.  de Savigny, Don, Professor; Health Systems and Policy Research, Swiss Tropical and Public Health Institute, Department of Epidemiology and Public Health, Switzerland
36.  de Wet, Katinka; Lecture, Sociology, The Humanities Faculty, University of the Free State, South Africa
37.  Delobelle, Peter; Senior Lecturer, School of Public Health, University of the Western Cape, South Africa
38.  Diaz, Theresa; Chief of Knowledge management for implementation Research UNICEF, United States of America
39.  Dieleman, Marjolein; Senior advisor Royal Tropical Institute Amsterdam and senior researcher Athena Institute Free University of Amsterdam, The Netherlands
40.  DiLiberto, Deborah D; PhD candidate, London School of Hygiene & Tropical Medicine, United Kingdom
41.  Dill, Leconte J; Assistant Professor, SUNY Downstate Medical Center, School of Public Health, United States of America
42.  Ditlopo, Prudence; Researcher, Centre for Health Policy, South Africa
43.  Doherty, Tanya; Chief Specialist Scientist, South African Medical Research Council, South Africa
44.  Erasmus, Ermin; Independent Health Policy and Systems Researcher, South Africa
45.  Fenenga, Christine; Senior Public Health Advisor/Senior Researcher, Learning and Analysis Unit, Amsterdam Institute for Global Health and Development (AIGHD) and Pharmaccess group, the Netherlands
46.  Flores, Walter; Executive Director, Center for the Study of Equity and Governance in Health Systems, Guatemala
47.  Foster, Nicola; Researcher, Health Economics Unit, University of Cape Town, South Africa
48.  Gautier, Lara; University of Montreal School of Public Health, Canada and Institute of Public Health Research, Paris-Diderot University, France
49.  Gebrehiwot, Tsegaye Tewelde; Lecturer, Jimma University, Ethiopia
50.  George, Asha; Professor, School of Public Health, University of the Western Cape, South Africa and Department of International Health, Johns Hopkins School of Public Health, United States of America
51.  Gideon, Jasmine; Senior Lecturer, Development Studies, Dept of Geography, Environment and Development Studies, Birkbeck, University of London, United Kingdom
52.  Gilson, Lucy; Professor, Health Policy and Systems Division, School of Public Health and Family Medicine, University of Cape Town, Department of Global Health and Development, London School of Hygiene and Tropical Medicine, South Africa
53.  Glenton, Claire; Senior Researcher, Global Health Unit, Norwegian Knowledge Centre, Norwegian Institute of Public Health, Norway
54.  Godt, Sue; Senior Programme Officer, International Development Research Centre (Canada), Regional office, Kenya
55.  Goicolea, Isabel; Associate Professor, Epidemiology and Global Health Unit, Department of Public Health and Clinical Medicine, Umeå University, Sweden
56.  Goma, Fastone M., Director, Centre for Primary Care Research, University of Zambia School of Medicine, Zambia
57.  Goudge, Jane; Director, Centre for Health Policy, University of the Witwatersrand, South Africa
58.  Govindasamy, Darshini; Senior scientist, Health Systems Research Unit, South African Medical Research Council, South Africa
59.  Gyselinck, Karel; Public Health Expert at BTC (Belgian Technical Cooperation) Health Team Brussels & Presidnet of Be-cause Health (Belgian Platform of International Health), Belgium
60.  Hammonds, Rachel; Post-Doctoral Researcher, Public Health Department, Institute of Tropical Medicine, Belgium
61.  Hanefeld, Johanna; Senior Lecturer Health Policy and Systems, Head Anthropology, Politics and Policy Group, London School of Hygiene and Tropical Medicine, United Kingdom
62.  Harris, Janet; NIHR Knowledge Mobilisation Research Fellow, Senior Lecturer, Course Director, International Health Management & Leadership, Course Director, Masters in Public Health (Management & Leadership), University of Sheffield, School of Health and Related Research, England
63.  Hickler, Benjamin; Health Specialist, UNICEF, United States of America
64.  Hipgrave, David; Senior Health Specialist, UNICEF, United States of America
65.  Ho, Lara S.; Senior Technical Advisor for Health Research, International Rescue Committee, United States of America
66.  Hoemeke, Laura; Director of Communications and Advocacy, IntraHealth International, United States of America
67.  Howard Natasha, Lecturer, London School of Hygiene & Tropical Medicine, United Kingdom
68.  Hurtig, Anna-Karin; Professor in Public Health, Unit of Epidemiology and Global Health, Umeå University, Sweden
69.  Jackson, Debra, Senior Health Specialist, UNICEF, United States of America
70.  Janse van Rensburg, André; Researcher, Centre for Health Systems Research & Development, University of the Free State; PhD candidate, Ghent University and Stellenbosch University, South Africa
71.  Jones, Caroline; position is Senior Social Scientist, Kemri-Wellcome Trust Research Programme, Kenya
72.  Juma, Pamela; African Population and Health Research Center, Kenya
73.  Kadidiatou, Kadio; Institutional affiliation, Doctoral Program in Applied Human Sciences (SHA), Université of Montréal, Canada, Institut de Recherche en Science de la Santé, Burkina Faso
74.  Kaim, Barbara; Programme Manager, Training and Research Support Centre, co-lead pra4equity learning network, EQUINET, Zimbabwe
75.  Kajja, Victoria; Masters student, Institute of Tropical Medicine, Belgium
76.  Kake, Amadou; NCDs program (Diabetes Unit), Ministry of Health, Guinea (Conakry)
77.  Kambala, Christabel Yollanda; PhD Student, Institute of Public Health, Heidelberg University, Germany School of Public Health and Family Medicine, College of Medicine, University of Malawi, Malawi
78.  Katz, Aaron; Principal Lecturer of Health Services, Global Health, and Law, School of Public Health, University of Washington, United States of America
79.  Keshri, Vikash R; Health Policy and System Researcher, Patna, India
80.  Khan, Kausar; Senior lecturer Aga Khan university, Dept of Community Health Sciences, Pakistan
81.  Kielmann, Karina; Senior Lecturer and Programme Leader, Institute for Global Health & Development Queen Margaret University, Scotland
82.  Kikule, Ekiria M. N.; Senior Lecturer & Head of Department (Public Health), Uganda Christian University, Uganda
83.  Knight, Lucia, Senior Lecturer, School of Public Health, University of the Western Cape, South Africa
84.  Koduah, Augustina; Position: Deputy programme manager, Institution: Ministry of Health, Ghana
85.  Koleva, Gergana; Patient Experience Researcher and Advocate for Patient and Public Involvement, Bulgaria
86.  Lakshmi, J. K.; Associate Professor, Indian Institute of Public Health, Hyderabad, Institution: Public Health Foundation of India, India
87.  Lanthorn, Heather; Senior Manager, IDinsight, India
88.  Lefèvre, Pierre; Senior Scientific Collaborator, Unit of Epidemiology and Disease Control, Department of Public Health, Institute of Tropical Medicine, Belgium
89.  Lehmann, Uta; Professor, School of Public Health, University of the Western Cape, South Africa
90.  Leon, Natalie; Specialist Scientist, South African Medical Research Council, South Africa
91.  Lesch, Anthea; Lecturer, Department of Psychology, Stellenbosch University, South Africa
92.  Lewin, Simon; Senior Researcher, Global Health Unit, Knowledge Centre for the Health Services, Norwegian Institute of Public Health, Oslo, Norway; Health Systems Research Unit, South African Medical Research Council, Cape Town, South Africa.
93.  Loewenson, Rene; Director, Training and Research Support Centre, Cluster lead EQUINET, East and Southern Africa
94.  London, Leslie; Professor of Public Health Medicine, University of Cape Town, South Africa
95.  Loveday, Marian; Specialist Scientist, Health Systems Research Unit, South African Medical Research Council, South Africa
96.  Macarayan, Erlyn Rachelle; Graduating PhD Student, The University of Queensland, Brisbane, Australia, Consultant, GAVI Vaccine Alliance, Geneva, Switzerland
97.  Machemedze, Rangarirai; Trade and Health programme Coordinator, SEATINI, Zimbabwe
98.  Machingura, Fortunate; Doctoral Student, University of Manchester - Global Development Institute, United Kingdom
99.  Magadzire, Bvudzai Priscilla, Researcher, School of Public Health, University of the Western Cape, South Africa
100.    Mamdani, Masuma; Chief Research Scientist, Ifakara Health Institute, Tanzania
101.    Marchal, Bruno; Department of Public Health, Institute of Tropical Medicine, Antwerp, Belgium
102.    Marten, Robert; Research Fellow in the Global Health and Development unit, London School of Hygiene and Tropical Medicine, United Kingdom
103.    Masquillier, Caroline; PhD Candidate, University of Antwerp, Belgium
104.    Mathews, Cathy; Director, Health Systems Research Unit, South African Medical Research Council, South Africa
105.    Mathews, Verona; Lecturer, School of Public Health, University of the Western Cape, South Africa
106.    Matthews, Anne; Head of School, School of Nursing & Human Sciences, Dublin City University, Ireland
107.    Mbakeh, Omar; Senior Planner, Ministry of Health and Social Welfare, The Gambia
108.    McIntyre, Di; South African Research Chair in Health and Wealth, Health Economics Unit, University of Cape Town, South Africa
109.    Meijering, Louise; Assistant Professor, University of Groningen, the Netherlands.
110.    Méndez, Claudio A.; Associate professor of health policy and systems,: Instituto de Salud Pública, Facultad de Medicina, Universidad Austral de Chile, Chile
111.    Mhatre, Sharmila; Program Leader, Maternal and Child Health, International Development Research Centre, Canada
112.    Michelo, Charles; Professor & Director, Department of Public Health, Medical School, University of Zambia, Zambia
113.    Mirzoev, Tolib; Associate Professor of International Health Policy and Systems, University of Leeds, United Kingdom
114.    Mishra, Arima; Associate Professor, Azim Premji University, India
115.    Mohammed, Shafiu; Health Systems and Policy Research Unit (HSPRU), Ahmadu Bello University, Zaria, Nigeria, Institute of Public Health, Heidelberg University, Germany
116.    Molyneux, Sassy; Associate Professor, University of Oxford and KEMRI-Wellcome Trust research programme, Kenya
117.    Msoka, Elizabeth Francis; Ass. Lecturer Kilimanjaro Christian Medical University College, Research Scientist at Kilimanjaro Clinical Research Institute, Kilimanjaro Clinical Research Institute (KCRI), Tanzania
118.    Mubangizi, Vincent; Lecturer/Family Medicine, Mbarara University of Science and Technology, Uganda
119.    Mugarura, Evatt M. (Rev); Executive Director, Africa Youth Leadership Development and Health Initiative, Uganda
120.    Mulumba, Moses; Executive Director, Centre for Health Human Rights and Development, Uganda
121.    Murray, Susan F; Professor of Health, Society and Development, International Development Institute, King’s College London, United Kingdom
122.    Nakkeeran, N., Associate Professor, Ambedkar University Delhi, India
123.    Namakhoma, Ireen; Executive Director, Research for Equity and Community Health Trust, Malawi
124.    Nambiar, Devaki; Public Health Researcher, India
125.    Neupane, Sunisha, PhD student, University of Montreal, Canada
126.    Noest, Stefan, Research Associate, Institution: University Hospital Heidelberg, Dept. of General Practice and Health Services Research, Germany
127.    Nxumalo, Nonhlanhla; Researcher & Deputy Director, Centre for Health Policy, School of Public Health, University of the Witwatersrand, South Africa
128.    Nyapada, Linet; Health Policy and Systems researcher, Kenya
129.    Nyström, Monica; Senior lecturer, LIME, Department of Learning, Informatics, Management and Ethics, MMC, Medical Management Center, Karolinska Institutet, Sweden
130.    Odendaal, Willem; Senior Scientist, Health Systems Research Unit, South African Medical Research Council, South Africa
131.    Olayo, Rose, Senior Lecturer/researcher, Jaramogi Oginga Odinga University of Science and Technology, Kenya
132.    Oliphant, Nicholas; Health Specialist, UNICEF, United States of America
133.    Olivier de Sardan, Jean Pierre, Professor, LASDEL, Niger
134.    Paina, Ligia; Assistant Scientist, Department of International Health, Johns Hopkins University School of Public Health, United States of America
135.    Paul, Mandira; Research Fellow, International Maternal and Child Health, Department of Women’s and Children’s Health, Uppsala University, Sweden
136.    Pérez, Dennis; Postdoc Researcher, Pedro Kouri Institute of Tropical Medicine in Havana, Cuba
137.    Prashanth, Nuggehalli Srinivas; Faculty, Institute of Public Health, India
138.    Probandari, Ari; Head of Public Health Department, Faculty of Medicine Universitas Sebelas Maret, Indonesia
139.    Rau, Asta; Director, Centre for Health Systems Research & Development, Faculty of Humanities, University of the Free State, South Africa
140.    Ridde, Valéry; Associate professor, IRSPUM/ESPUM, Canada
141.    Rodriguez, Daniela C.; Assistant Scientist, Johns Hopkins Bloomberg School of Public Health, United States of America
142.    Rosskam, Ellen; Principal, ER Global Consult, Switzerland
143.    Ruano, Ana Lorena; Centro de Estudios para la Equidad y Gobernanza en los Sistemas de Salud, Guatemala
144.    Sacks, Emma; Department of International Health, Johns Hopkins School of Public Health, Baltimore, USAID Maternal and Child Survival Program (MCSP)/ICF International, United States of America
145.    Sanders, David; Emeritus Professor, School of Public Health, University of the Western Cape, South Africa
146.    Scheel, Inger B.; Senior Researcher, Global Health Unit, Norwegian Knowledge Centre, Norwegian Institute of Public Health Norway
147.    Schleiff, Meike; Doctoral Candidate, Johns Hopkins Bloomberg School of Public Health, United States of America
148.    Schneider, Helen; Director & Professor, School of Public Health, University of the Western Cape, South Africa
149.    Scott, Kerry; Consultant,, India
150.    Scott, Vera; Senior Researcher, University of the Western Cape, South Africa
151.    Sharma, Reetu; Assistant Professor, Public Health Foundation of India, India
152.    Sheikh, Kabir; Senior Research Scientist, Public Health Foundation of India, India
153.    Shirin Singh, Namrita; Associate, Faculty, Department of International Health, Social & Behavioral Interventions, Site Director for Ukraine, USAID Victims of Torture Fund – JHU Institute for International Programs, Johns Hopkins Bloomberg School of Public Health, United States of America
154.    Srivatsan, R; Senior Fellow, Development and Health Initiative, Anveshi Research Centre for Women’s Studies, India
155.    Storeng, Katerini T.; Associate Professor, University of Oslo, Norway
156.    Swartz, Leslie; Professor, Department of Psychology/Departement  Sielkunde/Isebe LeSayikholoji, Stellenbosch University, South Africa
157.    Tetui, Moses; Research Fellow, Makerere University School of Public Health, Uganda; Umea International School Of Public Health, Umea University, Sweden
158.    Theobald, Sally; Professor in Social Science and International Health, Department of International Public Health, Liverpool School of Tropical Medicine, Liverpool; Institute of Development Studies, Sussex; United Kingdom
159.    Tolhurst, Rachel; Senior lecturer in Social Science and International Health, Department of Public Health, Liverpool School of Tropical Medicine, United Kingdom
160.    Topp, Stephanie M.; Senior Lecturer, College of Public Health, Medical and Veterinary Science, James Cook University, Australia
161.    Van Belle, Sara; post-doctoral fellow Health Policy Unit, Institution Institute of Tropical Medicine, Belgium
162.    van Heteren, Godelieve; Health Systems Reform and PBF expert, Director International Strategic Networks Erasmus University Rotterdam, The Netherlands
163.    van Zyl, André; Secretary of the Junior Public Health Association of South Africa, South Africa
164.    Wainwright, Megan; Postdoctoral Research Fellow, Faculty of Health Sciences, University of Cape Town, South Africa
165.    Wallerstein, Nina; Professor, Public Health Program, Director, Center for Participatory Research, Department of Family and Community Medicine, Senior Fellow, Robert Wood Johnson Center for Health Policy, University of New Mexico, United States of America
166.    Walt, Gill; Emeritus Professor of International Health, Global Health Development, Faculty Public Health and Policy, London School of Hygiene and Tropical Medicine, United Kingdom
167.    Walyaro, Connie; President, Citron Wood Foundation, Kenya
168.    Yaya Bocoum, Fadima; Chercheur, Institut de Recherche en Sciences de la Santé, Burkina Faso
169.    Zulu, Idah; Health Centre Incharge/Health literacy Coordinator/PAR facilitator, Lusaka District Health Office (Chaisa Health Centre), Zambia
170.    Zwi, Anthony; Professor of Global health and Development, Health Rights and Development, School of Social Sciences, The University of New South Wales, Australia
